# Procalcitonin neutralizes bacterial LPS and reduces LPS-induced cytokine release in human peripheral blood mononuclear cells

**DOI:** 10.1186/1471-2180-12-68

**Published:** 2012-05-08

**Authors:** Giovanni Matera, Angela Quirino, Aida Giancotti, Maria Concetta Pulicari, Linda Rametti, Maria Luz Rodríguez, Maria Carla Liberto, Alfredo Focà

**Affiliations:** 1 Institute of Microbiology, Department of Medical Sciences, University “Magna Graecia” of Catanzaro, I-88100, Catanzaro, Italy; 2 Randox Laboratories Limited, 5 Diamond Rd., Crumlin, County Antrim, BT29, 4QY, United Kingdom

## Abstract

**Background:**

Procalcitonin (PCT) is a polypeptide with several cationic aminoacids in its chemical structure and it is a well known marker of sepsis. It is now emerging that PCT might exhibit some anti-inflammatory effects. The present study, based on the evaluation of the *in vitro* interaction between PCT and bacterial lipopolisaccharide (LPS), reports new data supporting the interesting and potentially useful anti-inflammatory activity of PCT.

**Results:**

PCT significantly decreased (p < 0.05) the limulus amoebocyte lysate (LAL) assay reactivity of LPS from both *Salmonella typhimurium* (rough chemotype) and *Escherichia coli* (smooth chemotype). Subsequently, the *in vitro* effects of PCT on LPS-induced cytokine release were studied in human peripheral blood mononuclear cells (PBMC). When LPS was pre-incubated for 30 minutes with different concentrations of PCT, the release of interleukin-10 (IL-10) and tumor necrosis factor alpha (TNFα) by PBMC decreased in a concentration-dependent manner after 24 hours for IL-10 and 4 hours for TNFα. The release of monocyte chemotactic protein-1 (MCP-1) exhibited a drastic reduction at 4 hours for all the PCT concentrations assessed, whereas such decrease was concentration-dependent after 24 hours.

**Conclusions:**

This study provides the first evidence of the capability of PCT to directly neutralize bacterial LPS, thus leading to a reduction of its major inflammatory mediators.

## Background

The procalcitonin (PCT), the precursor for the hormone calcitonin (CT), is composed of 116-aminoacids and has a molecular weight of 13 kDa. PCT was discovered by Moya et al. in 1975, but its molecular structure was elucidated nine years later [1,2]. The primary structure of whole PCT includes some relevant polycationic motifs (2–3 bibasic aminoacids within a sequence of four) [1]. In sepsis, the marked increase of PCT concentration in serum has been reported [1,3].

The role of PCT as mediator of the sepsis cascade received much less attention. A pro-inflammatory activity of PCT in the pathogenesis of sepsis has been suggested based on immune-neutralization findings in two animal species [3]. An anti-inflammatory effect of PCT has been reported in very few studies [4-6], where the scarcity of the models/outcomes used does not lead to any firm conclusion. When human recombinant PCT was added to endotoxin-stimulated human whole blood, there was a marked decrease of the pro-inflammatory cytokine TNFα [5]. Interestingly, a reduction in IL-1β by administration of PCT was observed in the same animal model, the septic hamster, used for the first experiment of PCT immune-neutralization [6].

Lipopolysaccharide (LPS), the principal component of the outer leaflet of the outer membrane of Gram-negative bacteria, is recognized as the most potent microbial mediator implicated in the pathogenesis of sepsis sequelae and septic shock. Lipid A, the hydrophobic anchor of LPS, produces most of the responses after its detection by Toll-like receptor 4 (TLR-4).

Some LPS such as *Salmonella typhimurium* (*S. typhimurium)* LPS and *Escherichia coli* (*E. coli*) LPS*,* are well known endotoxins of rough and smooth chemotype [7]. Lipid A of *S. typhimurium* and *E. coli* LPS is a *β*1′-6-linked disaccharide of glucosamine, phosphorylated at the 1 and 4′ positions and acylated at the 2, 3, 2′, and 3′ positions with *R*-3-hydroxymyristate [8].

Therapeutic strategies for the treatment of septic shock in humans are currently focused on neutralization of the LPS molecule and its many deleterious effects [9].

Our previous investigations [10,11] and studies from other researchers [12], demonstrated that antimicrobial peptides, as well as other biological effective molecules sharing a polycationic structure [9], are able to neutralize LPS. Since PCT also presents two/three relevant polycationic motifs, comparable to some of the physical-chemical patterns of such antimicrobial peptides previously studied, we investigated the *in vitro* interaction between PCT and both rough and smooth chemotype LPS [7] by limulus amoebocyte lysate (LAL) test. As PCT was able to significantly decrease LAL assay reactivity in both LPSs tested, the effects of PCT-pre-incubated LPS on the release of cytokines in human peripheral blood mononuclear cells (PBMC) were examined. For this purpose, the mononuclear cell targeting chemokine (MCP-1), as well as Th1, Th2 and Treg type cytokines were selected.

## Results

### LPS-neutralizing activity of PCT

**Figure 1:**
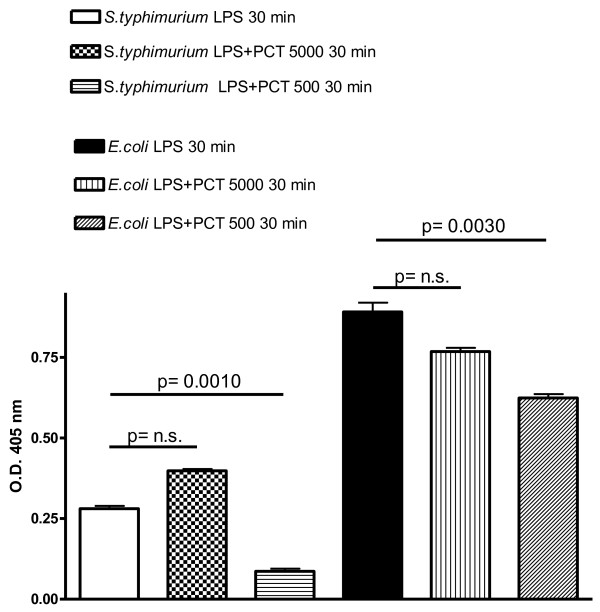
**Neutralization by PCT of LPS from *S. typhimurium *and *E. coli*.** The effect of PCT on *S. typhimurium* and *E. coli* LPS (100 pg/ml) reactivity was evaluated as O. D. (405 nm) by the chromogenic LAL test after 30 minutes incubation of the above reported LPS concentration: with 0 pg/ml PCT (LPS 30 min), with 5000 pg/ml PCT (LPS + PCT 5000 30 min), 500 pg/ml PCT (LPS + PCT 500 30 min). Results are presented as means ± SEM of at least four experiments each carried out in duplicate. Statistical significance between groups was assessed by Student’s *t* test. A *p* < 0.05 was considered significant, whereas not significant (n.s.) difference was associated with a p ≥ 0.05. Statistics were performed in comparison with respective LPS type-stimulated PCT-untreated cells (LPS 30 min), and the exact significance index is indicated on the top of the horizontal line encompassing the two statistically compared bars.

Following incubation of different concentrations of PCT with LPS for 30 minutes, PCT at a concentration of 500 pg/ml, significantly decreased the LAL reactivity of 100 pg/ml of both the rough LPS chemotype (*S. typhimurium* LPS, p = 0.0010) and the smooth LPS chemotype (*E. coli* LPS, p = 0.0030) (Figure 1)*.* Higher (5000 pg/ml) (Figure 1) or lower (50 pg/ml) (data not shown), concentrations of PCT did not produce any significant change in LAL reactivity of the LPS assessed.

### PCT effects on LPS-induced cytokine release

After 4 and 24 hours incubation of human PBMC with S*. typhimurium* LPS pre-incubated with PCT, the release of TNFα, IL-10, IL-4 and MCP-1 was simultaneously assessed with a cytokine biochip array.

LPS in RPMI 1640 medium in the absence of PCT induced a substantial increase of all the cytokines evaluated in human PBMC at both time points of 4 and 24 hours as expected.

**Figure 2:**
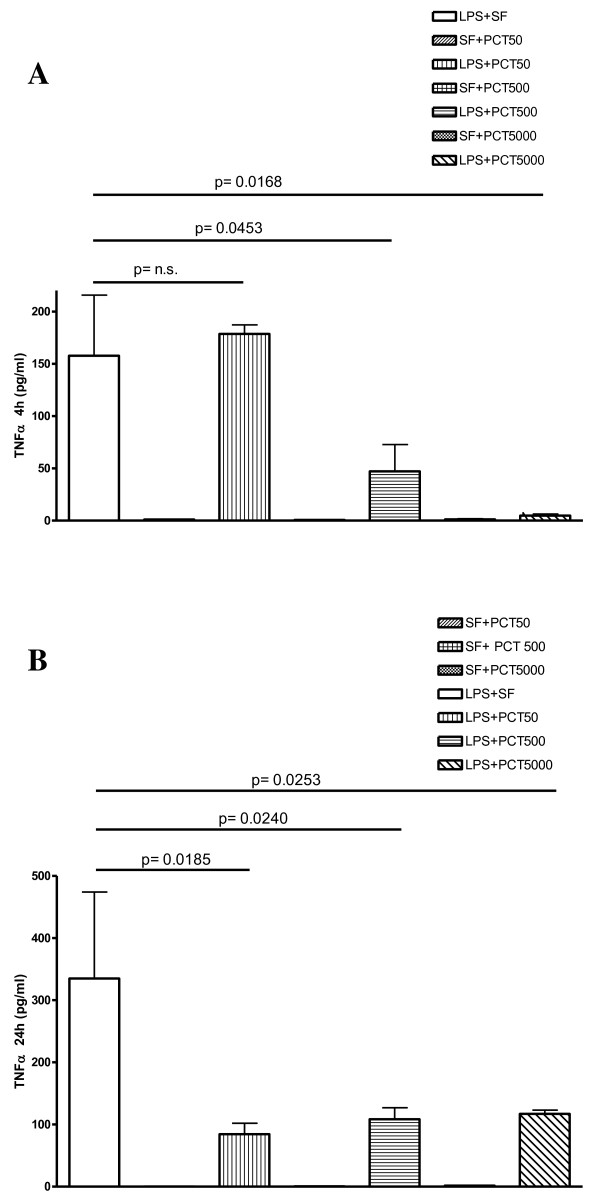
***In vitro *effect of different concentrations of PCT on *S. typhimurium *LPS-induced release of TNFα in human PBMC evaluated by cytokine biochip array.** Human PBMC were cultured for 4 h (panel A), and 24 h (panel B) with the following mixtures which had been pre-incubated at 37°C for 30 min : Sterile saline fluid (SF) plus 50 ng/ml PCT (SF + PCT 50); SF plus 500 ng/ml PCT (SF + PCT 500); SF plus 5000 ng/ml PCT (SF + PCT 5000); LPS of *S*. *typhimurium* SL1102 (100 ng/ml) plus SF (LPS + SF); LPS (100 ng/ml) plus 50 ng/ml PCT (LPS + PCT 50); LPS (100 ng/ml) plus 500 ng/ml PCT (LPS + PCT 500); LPS (100 ng/ml) plus 5000 ng/ml PCT (LPS + PCT 5000). Results are presented as means ± SEM of at least four experiments each carried out in duplicate. Statistical significance between groups was assessed by Student’s *t* test. A p < 0.05 was considered significant, whereas not significant (n.s.) difference was associated with a p ≥ 0.05. Statistics were performed in comparison with LPS-stimulated PCT-untreated cells (LPS + SF), and the exact significance index is indicated on the top of the horizontal line encompassing the two statistically compared bars.

When LPS was pre-incubated with PCT at different concentrations, a decrease of the TNFα release was observed for both time points, this reduction was concentration-dependent at 4 hours (Figure 2). The LPS-induced release of TNFα after 4 hours of incubation was significantly reduced by 500 ng/ml (p = 0.0453) and by 5000 ng/ml (p = 0.0168) of PCT in comparison to LPS plus saline-treated PBMC.

Following 24 hours of incubation, TNFα release stimulated by LPS was significantly diminished when PCT was used at 50 (p = 0.0185), at 500 (p = 0.0240) and at 5000 ng/ml (p = 0.0253).

**Figure 3:**
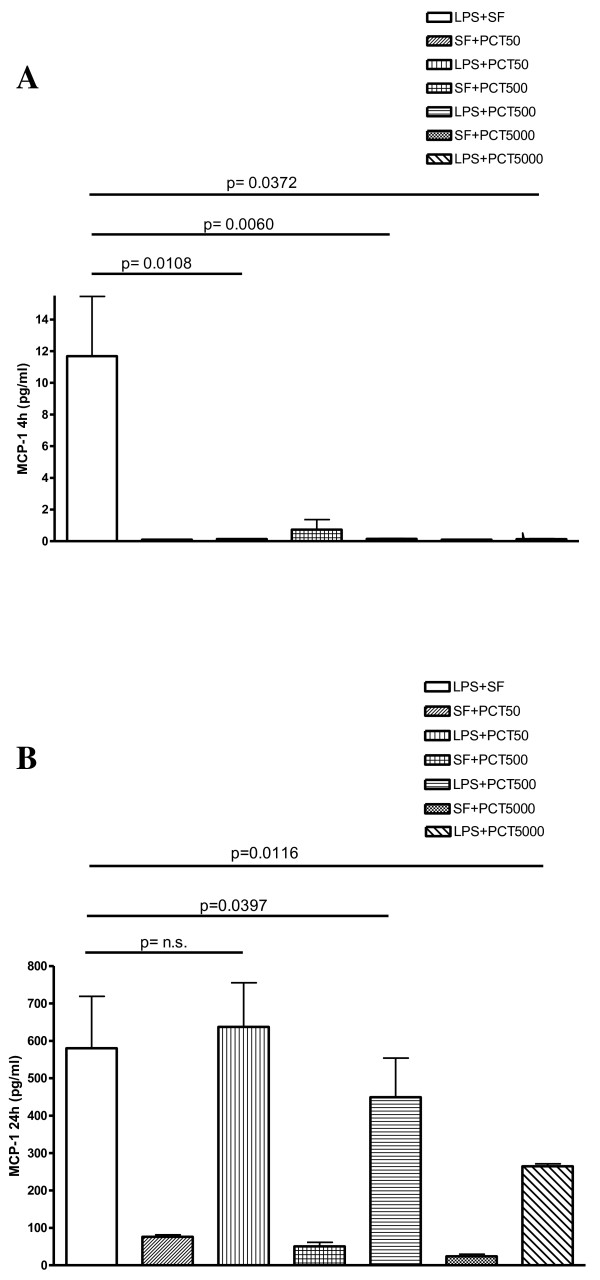
***In vitro *effect of different concentrations of PCT on *S. typhimurium *LPS-induced release of MCP-1 evaluated by cytokine biochip array.** Human PBMC were cultured for 4 h (panel A), and 24 h (panel B) with the following mixtures which had been pre-incubated at 37°C for 30 min : Sterile saline fluid (SF) plus 50 ng/ml PCT (SF + PCT 50); SF plus 500 ng/ml PCT (SF + PCT 500); SF plus 5000 ng/ml PCT (SF + PCT 5000); LPS of *S*. *typhimurium* SL1102 (100 ng/ml) plus SF (LPS + SF); LPS (100 ng/ml) plus 50 ng/ml PCT (LPS + PCT 50); LPS (100 ng/ml) plus 500 ng/ml PCT (LPS + PCT 500); LPS (100 ng/ml) plus 5000 ng/ml PCT (LPS + PCT 5000). Results are presented as means ± SEM of at least four experiments each carried out in duplicate. Statistical significance between groups was assessed by Student’s *t* test. A p < 0.05 was considered significant, whereas not significant (n.s.) difference was associated with a p ≥ 0.05. Statistics were performed in comparison with LPS-stimulated PCT-untreated cells (LPS + SF), and the exact significance index is indicated on the top of the horizontal line encompassing the two statistically compared bars

**Figure 4:**
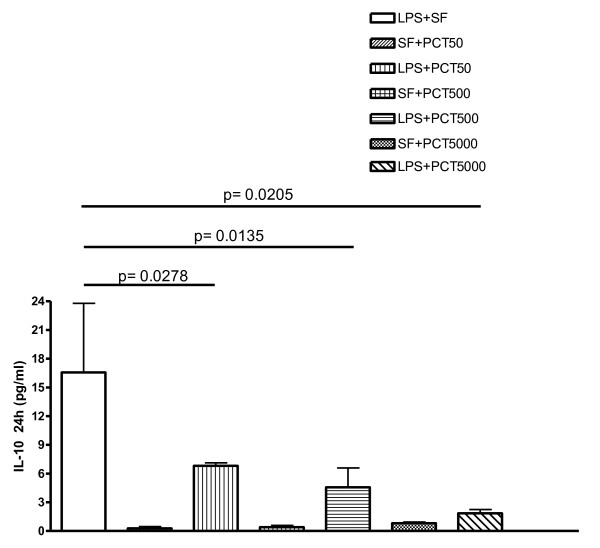
***In vitro *effect of different concentrations of PCT on *S. typhimurium *LPS-induced release of IL-10 evaluated by cytokine biochip array.** Human PBMC were cultured for 24 h with the following mixtures which had been pre-incubated at 37°C for 30 min: Sterile saline fluid (SF) plus 50 ng/ml PCT (SF + PCT 50); SF plus 500 ng/ml PCT (SF + PCT 500); SF plus 5000 ng/ml PCT (SF + PCT 5000); LPS of *S*. *typhimurium* SL1102 (100 ng/ml) plus SF (LPS + SF); LPS (100 ng/ml) plus 50 ng/ml PCT (LPS + PCT 50); LPS (100 ng/ml) plus 500 ng/ml PCT (LPS + PCT 500); LPS (100 ng/ml) plus 5000 ng/ml PCT (LPS + PCT 5000). Results are presented as means ± SEM of at least four experiments each carried out in duplicate. Statistical significance between groups was assessed by Student’*t* test. A *p* < 0.05 was considered significant. Statistics were performed in comparison with LPS-stimulated PCT-untreated cells (LPS + SF), and the exact significance index is indicated on the top of the horizontal line encompassing the two statistically compared bars.

The levels of MCP-1 were drastically reduced after 4 hours for all the PCT concentrations (Figure 3A). Moreover after 24 hours, the MCP-1 release significantly decreased following both 500 (p = 0.0397) and 5000 ng/ml (p = 0.0116) of PCT (Figure 3B). In the same experimental setting, the LPS-stimulated release of IL-10 showed a dose-dependent inhibition by PCT at 24 h that was significant at a concentration of 50 (p = 0.0278), 500 (p = 0.0135) and 5000 ng/ml (p = 0.0205) of the polypeptide (Figure 4). After 4 hours, this cytokine exhibited slower kinetic. Even though the release of IL-10 by PCT/LPS-incubated PBMC was significantly (p < 0.05) lower than in the supernatant of LPS alone-challenged PBMC, the level of this cytokine was still quite low and perhaps not biologically relevant (data not shown).

The release of IL-4 was not affected by PCT (data not shown).

Direct assay (trypan blue test and acridine orange vital staining) of cellular viability always indicated a percentage of more than 95% viable cells in any experimental group, even after 24 h of PBMC incubation, which would indicate that the observed reduction in cytokine release may not be due to cellular toxicity by PCT, LPS or both. Also cell count was carried out at beginning and at the end of each experiment and these values were not significantly different. Therefore a decrease of cell number should be excluded as a possible cause of reduced cytokine release, during the experiments which involved PCT.

## Discussion

The main and novel findings of the present study are the PCT-induced decrease of bacterial LPS reactivity and the reduction of LPS- induced release of some cytokines/chemokines by PCT in human PBMC. Previous studies from our group [10,11] and from other investigators [12], demonstrated that antimicrobial peptides (teicoplanin and magainins) and other biological effective molecules presenting a polycationic structure, can neutralize both the LAL reactivity and other effects of LPS including cytokine release [9,13].

An examination of the PCT primary structure reveals that relevant polycationic motifs (sequence of at least 2–3 bibasic aminoacids within a sequence of four) are present in the whole molecule. Therefore, the whole PCT molecule may account for binding and neutralizing the LPS as well as inhibiting the LPS-stimulated mediators.

Other structural studies [14] produced a more direct evidence that neutralization of LPS follows interactions between on one hand, positively charged and hydrophobic groups of the peptide and on the other hand, phosphates and hydrophobic acyl chains of the conserved lipid A moiety. It has been demonstrated that in the LPS-neutralizing peptide, the lipid A binding motif includes a cluster of hydrophobic residues encompassed by basic aminoacids [14].

**Figure 5:**
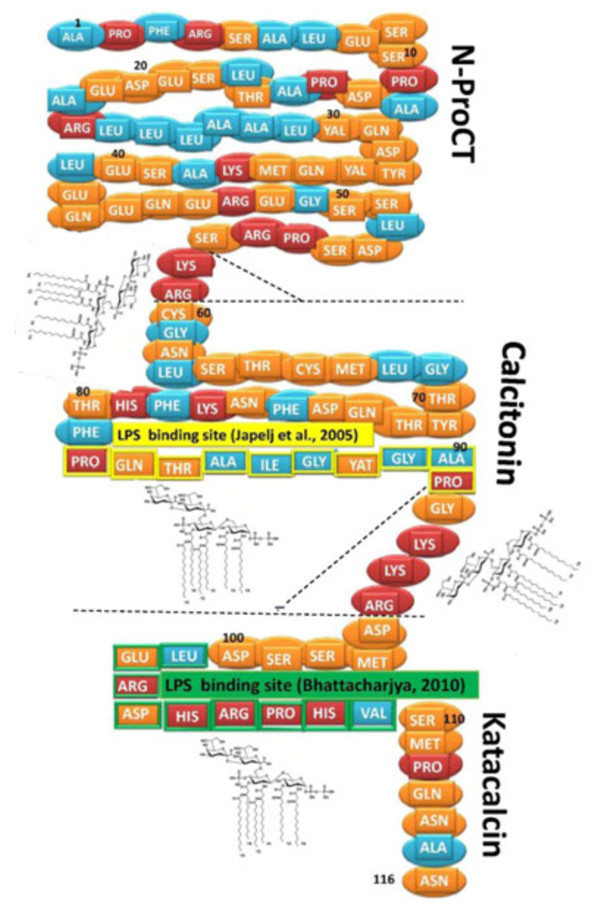
**Putative LPS binding sites on PCT molecule.** Proposed LPS binding sites include: i) 2–3 cationic aminoacids within a cluster of four (aminoacids 58–59 and aminoacids 93–95), ii) a cluster of hydrophobic residues encompassed by basic aminoacids (82–92), iii) a group of positively charged central residues with hydrophobic aminoacids in the periphery (101–109). Hydrophobic aminoacids in blue, cationic aminoacids in red and other aminoacids in orange. The LPS binding sites suggested by Japelj [14] and Bhattacharjya [15] are indicated. Close to the proposed LPS binding sites, a deep rough LPS chemical structure is showed. Flat dashed lines indicate the limits of the three post-translational processing products (N-ProCT, calcitonin and katacalcin) of procalcitonin, while dashed forks encompass the peptides cleaved during post-translational processing [1,3].

More recently, other authors underlined the pivotal role of a group of positively charged central residues with hydrophobic aminoacids distributed in the periphery [15]. The whole PCT used in our study, exhibited a plausible lipid A binding sequence between Pro^82^ and Pro^91^[14]. Also a putative lipid A binding sequence can be found between Leu^101^ and Val^109^[15] as illustrated in Figure 5.

It has also been reported that the need for structural amphipathicity is probably not as an essential feature for LPS binding/neutralization as is the proximity of certain aminoacids (cationic and hydrophobic residues) within a given sequence [16].

The effects of PCT on LPS reactivity in the LAL test model suggest that PCT is equally active against both rough and smooth chemotypes. The *S. typhimurium* strain SL1102 exhibits a Re chemotype LPS (deep rough) that has been previously reported as very toxic in an *in vivo* experimental model [17]. The *E. coli* 0111:B4 has a smooth chemotype endotoxin often used in studies regarding LPS binding/neutralization [18]. Therefore PCT targets the lipid A portion which is a common structural feature of these LPSs.

Since the molecular weight of PCT is approximately 13,000 daltons and the molecular weight of deep rough LPS is 3,000 daltons, the optimal ratio 5:1 (w/w) associated with LPS neutralization and cytokine inhibition would suggest a 1mole:1mole interaction between PCT and LPS, which could use any of the above mentioned interaction sites available on the PCT molecule.

Moreover, our results provide the first evidence of the capability of PCT to significantly decrease the LPS-stimulated release of the Treg cytokine IL-10 and chemokine MCP-1 from human PBMC. The PCT-induced decrease in the release of TNFα found with this experimental model, agrees with the findings reported using a different *in vitro* approach [5].

The late and significant decrease of LPS-stimulated IL-10 may suggest a clinically valuable role of PCT in the control of this cytokine during late stages of sepsis, often associated with immunoparalysis, when IL-10 is reported to play a pivotal role [19,20].

PCT and/or its fragment (e.g. N-PCT) have been shown to cause some anti-inflammatory effects in some experimental models [4]. In contrast, Becker et al. [3] reported that PCT produced only detrimental effects in the host.

According to our data and data from other investigators [5,21], in clinical/experimental sepsis the large amount of TNFα production and its detrimental effects for the host may be controlled by PCT release. Unlike TNFα, which mimics most of the LPS-induced signs and symptoms of the sepsis [19], PCT did not show any detrimental effects when injected in healthy animals [3,22] even at high dose. Moreover, in septic hamster serum TNFα concentration was not affected by PCT administration, which was able to significantly decrease IL-1β serum level [6].

A very recent publication on the *in vitro* effect of PCT on whole blood from healthy humans revealed that most of the cytokines evaluated in the supernatant were not affected by PCT. Only IL-6 exhibited a substantial increase; whereas TNFα increased to a lesser extent and IL-13 was significantly reduced by PCT. Human neutrophils challenged *in vitro* with several concentrations of PCT did not significantly change cytokine release [23].

In human monocytes endogenous TNFα is crucial for subsequent IL-10 synthesis through autocrine and paracrine mechanisms [24]. Therefore, reduction of TNFα levels by PCT may supposedly result in decreased IL-10 synthesis. Wiedermann et al. [25] reported that PCT was able to decrease migration of monocytes towards different chemoattractants including MCP-1. Moreover, N-PCT has been found to reduce the expression of CD11b, a major integrin involved in monocyte chemotaxis mechanism. Our data suggest a novel aspect of the PCT-mediated control on monocyte chemotaxis, with a direct decrease of LPS-induced MCP-1 by PCT. Based on our results, in the presence of PCT, multiple mechanisms would modulate monocyte chemotaxis, reducing systemic inflammatory host response, which might follow exaggerated activation of phagocytes during sepsis [26].

Cellular toxicity of PCT, LPS or PCT plus LPS should not account for cytokine reduction by PCT, because the direct assays of cell viability always indicated a percentage of living cells higher than 95%, even after 24 hours of incubation. Moreover, studied cytokines would be expected to show substantial changes (due to cytotoxicity) with addition of PCT alone, but this was not the case. The increase of MCP-1 released by PBMC induced by LPS is ten to twenty-fold higher than in PCT-stimulated PBMC. The latter is not significantly different when compared to unstimulated PBMC incubated for 24 h (data not shown). Even more importantly, the highest release of MCP-1 is associated to the lowest concentration of PCT. Also cell count was carried out at beginning and at the end of each experiment and these values were not significantly different. Therefore a decrease of cell number should be excluded as a possible cause of reduced cytokine release, during the experiments which involved PCT.

Despite the interest and novelty of the present findings, the LPS neutralization might be only one of the major modulatory mechanisms of PCT on “cytokine storm” during sepsis. As the present study is based on an in vitro model, some limitations regarding the drawing of more general conclusions, the extrapolation to the in vivo activity and the potential role of PCT in the therapy of systemic inflammatory diseases are acknowledged.

## Conclusions

In conclusion our data indicate a direct LPS neutralizing effect of PCT, which suggests a significant PCT-induced inhibition on major mediators of the Th1, Treg and monocyte activation cascade stimulated by LPS. Any agent, including PCT, with the capability to neutralize an early stimulus such as a bacterial product (e.g. LPS) and reduce the release of sepsis mediators deserves further investigation. These reported findings may provide new insights into biological and clinical events of the physiopathology of sepsis.

## Methods

### Chemicals

The LPS of *E. coli* strain O111:B4 was from Cambrex (Walkersville, USA); the LPS of *S. typhimurium* strain SL1102 was extracted and purified as previously described [17]. Recombinant human procalcitonin was a generous gift of Randox (Randox Laboratories Ltd., Crumlin, UK). RPMI 1640 medium was obtained from Invitrogen (Carlsbad, CA).

### LAL test

For the evaluation of the LPS-neutralizing activity of PCT, LPS from *S. typhimurium* and *E. coli* were dissolved in sterile water for injection and then diluted in apyrogenic saline fluid (SF). Serial dilutions of PCT (5000, 500 and 50 pg/ml) in SF were incubated with 100 pg/ml of LPS from *S. typhimurium* and *E. coli* in a sterile conic tube at 37°C for 30 min. In preliminary experiments the reactivity of *S. typhimurium* and *E. coli* LPS was tested at different time points following LPS-PCT co-incubation. An incubation time of 30 min was found to be optimal based on higher LPS reactivity in the LAL test and more obvious PCT effect on such reactivity (Quirino A. personal observation). The LPS-neutralizing activity of PCT was analyzed using the chromogenic LAL-test (QCL-1000, Cambrex, Walkersville, USA) following manufacturer’s instructions, but the results were reported as optical density (O.D.) at 405 nm and were not corrected for the dilution factor [10].

### PBMC stimulation

For the study of the effects of PCT-pre-incubated LPS in cytokine release, human PBMC were obtained from blood samples of healthy donors, who gave informed consent.

All procedures were conducted in accordance with the guidelines of the local ethics committee at the Medical Faculty of the University “Magna Graecia” of Catanzaro, which are in compliance with Declaration of Helsinki (59th WMA General Assembly, Seoul, October 2008).

Cells were isolated from heparinized whole blood by Ficoll (Ficoll-Paque, SIGMA, Italy) density gradient purification technique. After washing with PBS and counting, the cells were resuspended in RPMI 1640 medium in the absence of antibiotics and glutamine. The cells were then incubated in 24-well flat bottom tissue culture plates (Falcon, Becton Dickinson Labware, Franklin Lakes, New Jersey) at a final concentration of 1.5 × 10^5^ cells/ml for 4 and 24 hours with LPS of *S*. *typhimurium* SL1102 (100 ng/ml). The latter was previously incubated for 30 min with different concentrations of PCT (5000-500-50 ng/ml). Cells incubated with the same PCT concentrations in absence of LPS and cells incubated with LPS in absence of PCT, were used as controls.

The cytotoxicity of PCT, LPS and PCT plus LPS was tested by trypan blue test (11) and by acridine orange vital staining, after both 4 and 24 h of PBMC incubation. In all cases the percentage of viable cells was higher than 95%. Also cell count was carried out at beginning and at the end of each experiment and these values were not significantly different.

Supernatants from PBMC cultures were collected and assayed for simultaneous determination of Th1, Th2 and Treg cytokines using a cytokine biochip array on the Evidence Investigator analyser following the manufacturer’s instructions (Randox Laboratories Ltd., Crumlin, UK). For this study data on IL-10, IL-4, TNFα and MCP-1 were evaluated.

### Statistical analysis

Statistical significance between groups was assessed by the Student’s *t* test. Results were presented as means ± SEM of at least four experiments each carried out in duplicate. A *p* value <0.05 was considered to be statistically significant.

## Abbreviations

PCT: Procalcitonin; LPS: Lipopolysaccharide; LAL: Limulus amoebocyte lysate; PBMC: Peripheral blood mononuclear cell; IL-10: Interleukin-10; TNFα: Tumor necrosis factor alpha; MCP-1: Monocyte chemotactic protein-1; CT: Calcitonin; TLR-4: Toll-like receptor 4; S. typhimurium: Salmonella typhimurium; E. coli: Escherichia coli; SF: Saline fluid; O.D: Optical density; SEM: Standard error of the mean.

## Competing interests

Financial support for this research was entirely provided by the University of Catanzaro. M.L. Rodríguez is an employee of Randox Laboratories Limited.

## Authors’ contributions

GM conceived the study, drafted the manuscript and participated in its design. AQ carried out PBMC experiments, contributed to the LAL experiments and participated in the draft of the manuscript. AG carried out LPS neutralizing test by LAL. MCP contributed to the LAL studies, PBMC experiments and performed statistical analysis. LR contributed to LAL test and carried out cytokine biochip array analysis. MLR participated in the draft and editing of the manuscript. MCL participated in the design and coordination of the study and contributed in the draft and editing of the manuscript. AF conceived the study and participated in its design and coordination. All authors read and approved the final manuscript.
